# Liquid and vapour-phase antifungal activities of essential oils against *Candida albicans* and non*-albicans Candida*

**DOI:** 10.1186/s12906-016-1316-5

**Published:** 2016-08-30

**Authors:** Narcisa Mandras, Antonia Nostro, Janira Roana, Daniela Scalas, Giuliana Banche, Valeria Ghisetti, Simonetta Del Re, Giacomo Fucale, Anna Maria Cuffini, Vivian Tullio

**Affiliations:** 1 Department of Public Health and Paediatrics, Microbiology Division, University of Turin, via Santena 9, 10126 Turin, Italy; 2 Department of Pharmaceutical Sciences and Health Products Polo Annunziata, University of Messina, Viale S.S. Annunziata, 98168 Messina, Italy; 3 Laboratory of Microbiology and Virology, Amedeo di Savoia Hospital, c.so Svizzera 164, 10149 Torino, Italy; 4 Chemical, Clinical and Microbiological Analyses Department, CTO, Turin, Italy

**Keywords:** Antifungal activity, Yeasts, Essential oils, Broth microdilution method, Vapour contact assay

## Abstract

**Background:**

The management of Candida infections faces many problems, such as a limited number of antifungal drugs, toxicity, resistance of Candida to commonly antifungal drugs, relapse of Candida infections, and the high cost of antifungal drugs. Though azole antifungal agents and derivatives continue to dominate as drugs of choice against Candida infections, there are many available data referring to the anticandidal activity of essential oils. Since we have previous observed a good antimicrobial activity of some essential oils against filamentous fungi, the aim of this study was to extend the research to evaluate the activity of the same oils on *Candida albicans, C.glabrata* and *C.tropicalis* clinical strains, as well as the effects of related components. Essential oils selection was based both on ethnomedicinal use and on proved antibacterial and/or antifungal activity of some of these oils. Fluconazole and voriconazole were used as reference drugs.

**Methods:**

The minimum inhibitory concentration (MIC) and the minimal fungicidal concentration (MFC) of essential oils (thyme red, fennel, clove, pine, sage, lemon balm, and lavender) and their major components were investigated by the broth microdilution method (BM) and the vapour contact assay (VC).

**Results:**

Using BM, pine oil showed the best activity against all strains tested, though *C.albicans* was more susceptible than *C.glabrata* and *C.tropicalis* (MIC_50_-MIC_90_ = 0.06 %, v/v). On the contrary, sage oil displayed a weak activity (MIC_50_-MIC_90_ = 1 %, v/v). Thyme red oil (MIC_50_-MIC_90_ ≤ 0.0038 %, v/v for *C.albicans* and *C.tropicalis*, and 0.0078- < 0.015 %, v/v for *C.glabrata*), followed by lemon balm, lavender and sage were the most effective by VC. Carvacrol and thymol showed the highest activity, whereas linalyl acetate showed the lowest activity both by two methods. α-pinene displayed a better activity by BM than VC.

**Conclusion:**

Results show a good activity of essential oils, mainly thymus red and pine oils, and their components carvacrol, thymol and α-pinene against *Candida* spp., including fluconazole/voriconazole resistant strains. These data encourage adequately controlled and randomized clinical investigations. The use in vapour phase could have additional advantages without requiring direct contact, resulting in easy of environmental application such as in hospital, and/or in school.

## Background

Candidiasis is the most common opportunistic yeast infection and encompasses infections that range from superficial mucosal infections, such as oral thrush and vaginitis, to systemic and potentially life-threatening diseases, such as disseminated candidiasis. In the last two decades, it has been observed a considerable increase in the incidence of deep fungal infections, not only in immunocompromised patients related to nosocomial infections, but also in healthy subjects. Moreover, the incidence of *C. albicans*, the leading pathogenic Candida species so far, has declined while that of non-*albicans Candida* is increased [1].

The most commonly used classes of antifungal agents to treat Candida infections are the azoles, polyenes, and echinocandins; however, the management of Candida infections faces many problems, such as toxicity, resistance of Candida to commonly used antifungal drugs, relapse of Candida infections, and the high cost of antifungal drugs [2, 3]. To elude these problems, investigators are exploiting alternative therapeutic strategies, such as the use of natural products, especially essential oils (EOs) [4–7].

EOs have long been used in ethnomedicine as effective and safe antifungal agents; however, good scientific and clinical data that either supports or contravenes the effectiveness of these alternative therapies are still needed before consumers can be sure they will enjoy any benefits. Previously we have studied the antimicrobial activity of thyme red, clove, pine, sage, lemon balm, fennel, lavender EOs against filamentous fungi [8]. Hence, the objective of this study was to extend the research to evaluate the activity of the same EOs on *Candida albicans* and non-*albicans Candida* strains, as well as the effects of related EO components, by using two investigative tools, such as the broth microdilution method (BM) and the vapour contact assay (VC). EOs selection was based both on ethnomedicinal use and on proved antibacterial and/or antifungal activity of some of these oils [8, 9]. Fluconazole and voriconazole were used as reference drugs to compare EOs activity.

## Methods

### Essential oils and their components

The EOs have directly been purchased from Azienda Agricola Aboca (Sansepolcro, Arezzo) as steam distilled samples obtained from *Thymus vulgaris* L. (thyme red), *Foeniculum vulgare* Mill. var. *dulce* DC (fennel), *Eugenia caryophyllata* Thumb. (clove), *Pinus sylvestris* L. (pine), *Salvia officinalis* L. (sage), *Melissa officinalis* L. (lemon balm) and *Lavandula vera* DC (lavender). The components carvacrol, eugenol, linalool, linalyl acetate, thymol and α-pinene (≥98 % purity) were supplied by Sigma Aldrich (Steinheim, Germany) and used as received without any further purification. EOs and related components were storage at 4 °C until use.

### Essential oils and their components stock solutions

Stock solutions of each EO and its components were prepared in ethanol (1:25) and diluted (1:20) to obtain a final concentration of 2 % (v/v) in RPMI-1640 without sodium bicarbonate and with L-glutamine (Invitrogen, San Giuliano Milanese, Milano, Italy), buffered to pH 7.0 with 0.165 M morpholinepropanesulfonic acid (MOPS) (Sigma) at a concentration of 0.165 mol 1^−1^ and supplemented with glucose 18 g/L. To enhance EOs solubility, Tween-80 (Sigma-Aldrich) was included at a final concentration of 0.001 % (v/v).

### Antifungal drugs

Fluconazole and voriconazole powders (≥98 % purity by HPLC) were purchased from Sigma-Aldrich (n° F8929 and PZ0005, respectively). Fluconazole stock solutions were prepared in sterile distilled water, while voriconazole in 100 % dimethyl sulfoxide (Sigma-Aldrich), and stored at −20 °C until use.

### Yeasts

Forty-six yeasts, including *C. albicans* (*n* = 26), *C. glabrata* (*n* = 10) and *C. tropicalis* (*n* = 10) were collected from various specimens (blood, normally sterile body fluids, deep tissue, genital tract, gastrointestinal tract, respiratory tract) from hospitalized patients in Turin (Italy). The strains were identified by standard methods (cornmeal for blastoconidia, germ-tube formation, pseudohyphae and true hyphae, and growth on CHROMagar™ Candida (BD, Milan, Italy) and with commercially available yeast identification system (API ID32C panels, bioMérieux, Rome, Italy) [10].

### Inoculum preparation

Yeasts inocula were prepared by picking two to three colonies of >1 mm diameter from an overnight culture of *Candida* spp. on Sabouraud dextrose (SAB) agar at 35 °C, and suspending them in 2 mL of 0.85 % normal saline, to yield a yeast stock suspension of ≈ 5 × 10^6^ cells/mL by 0.5 McFarland standard. A working suspension was made by a 1:100 dilution followed by a 1:20 dilution of the stock suspension with RPMI 1640 broth medium (0.2 % glucose) (Sigma, Milan, Italy), which resulted in ≈ 2.5 × 10^3^ cells/mL, confirmed by colony counts in triplicate. The cell density was adjusted to 2.0 × 10^3^ cells/mL, confirmed by colony counts in triplicate.

### In vitro antimicrobial assays

#### Broth microdilution method

The antimicrobial activity of EOs and their components was determined using a BM susceptibility assay, according to CLSI document M27-A3 for yeasts with some modifications [11]. Minimum inhibitory concentration (MIC) determination was performed by serial dilution using 96-well microtitre plates (Sarstedt, Milan, Italy). Doubling dilutions of the EOs ranging from 2 to 0.0038 % (v/v) were prepared in 96-well microtitre trays in RPMI-1640 with MOPS buffered to pH 7. Furthermore, each yeast strain included in the study was tested for its sensitivity to fluconazole and voriconazole following the M27-A3 protocol [11]. Doubling dilutions of the two reference antifungal compounds, ranging from 128 to 0.008 μg/mL, were prepared in 96-well microtitre trays in RPMI-1640 with MOPS buffered to pH 7. After inoculum addition (0.1 mL), the trays were incubated under normal atmospheric conditions at 37 °C for 48 h. A sterile medium incubated under the same condition was used as a blank, while the medium inoculated with the target microorganisms (without the oil/drug) was used as a positive control of growth. All determinations were performed in triplicate.

The lowest concentration of the oil showing complete inhibition of visible growth was defined as MIC. The absence of visible growth was determined under a binocular microscope. MICs of azoles were read as the lowest drug concentration that produced ≥50 % growth inhibition in comparison with growth control. The CLSI resistance breakpoint for fluconazole was defined as a MIC of ≥ 2 μg/mL against *C. albicans*, *C. tropicalis* and a MIC of ≥32 μg/mL against *C. glabrata*; the CLSI resistance breakpoint for voriconazole was defined as a MIC of ≥ 0.5 μg/mL against *C. albicans* and *C. tropicalis*. CLSI has not assigned breakpoints for voriconazole and *C. glabrata*, and recommended the epidemiological cut-off value (ECV) of 0.5 μg/mL to be used to differentiate wild type (WT) from non-WT strains of this species [12].

The minimal fungicidal concentration (MFC) of EOs, their components, and drugs was determined by subculturing 10 μL of broth taken from all the wells without visible growth onto SAB agar plates that do not contain the test agents. After incubation for 48 h at 37 °C, MFC was defined as the lowest concentration of oil/drug resulting in the death of 99.9 % of the inoculum in no growth on subculture [8, 13].

#### Vapour contact assay

The effect of volatile oil fraction was studied with invert Petri dishes method as previously reported [8, 14]. Double-strength concentrated RPMI 1640 with MOPS, adjusted to pH 7.0 was mixed with molten 3.0 % (w/v) agar in an equal ratio immediately before the assay. The RPMI agar was poured into a 90 mm Petri dish and spot inoculated with 100 μL of standardized suspension of each strain tested. A glass slide (1-cm size) was placed in the cover of each Petri dish, so that it did not directly touch the surface of the agar medium, and various amounts of pure EOs or their components were added to obtain final concentration ranging from 1 to 0.0019 %, v/v air space. The space inside of the sealed Petri dish was calculated to be 70 cm^3^ air. The plates were sealed with vinyl tape immediately after inoculation, and incubated at 37 °C for 48 h. The control, consisting of RPMI medium with MOPS, was included. The MIC (percentage, v/v air space) was determined by comparison with the control and was defined as the lowest concentration of EO inhibiting the visible growth.

### Data analysis

The data from at least three replicates were evaluated and modal results were calculated.

## Results and discussion

The research on EOs and closely related components has been recently intensified, due to their biological, antioxidant and antimicrobial properties [5, 15–18]. Moreover, there is a growing evidence that EOs in vapour phase are effective antimicrobial systems and that they could have advantages over the use of EOs in liquid phase, especially in a hospital environment. In fact, our previously studies demonstrated a better activity in vapour phase of some oils (thyme red, fennel, clove, pine, sage, lemon balm and lavender) against human and plant pathogen filamentous fungi [8].

In this study, we evaluated by two methods (BM and VC) the antifungal activity of seven EOs including thyme red, fennel, clove, pine, sage, lemon balm and lavender (Table 1) and some their components (Table 2), towards 46 clinical isolates of *C. albicans*, *C. glabrata* and *C. tropicalis*. In literature, there are some available data referring to the anticandidal activity of EOs such as thyme, clove, pine, fennel, sage, and lemon balm [5–7, 19, 20]. 

Our results, reported as concentrations of EO, major components, fluconazole and voriconazole, are showed as MIC_50_ (Minimum Inhibitory Concentration required to inhibit the growth of 50 % of yeasts) and MIC_90_ (Minimum Inhibitory Concentration which inhibits the growth of 90 % of yeasts), respectively (Tables 1, 2).

**Table 1:** Minimum inhibitory concentration (MIC) of EOs (%, v/v) and drugs (μg/mL) against *Candida spp.* evaluated by the broth microdilution (BM) and vapour contact (VC) methods

Essential oils/drugs	Methods	*C.albicans* (*n* = 26)			*C.glabrata* (*n* = 10)			*C.tropicalis* (*n* = 10)		
		Range	MIC 50	MIC 90	range	MIC 50	MIC 90	range	MIC 50	MIC 90
Thyme red	BM	0.03–0.25	0.03	0.125	0.06–0.25	0.06	0.125	0.06–0.25	0.06	0.25
	VC	<0.0038	<0.0038	<0.0038	<0.0019–0.03	0.0078	0.015	0.0075–0.015	0.0038	0.0038
Fennel	BM	0.25–1	0.25	1	0.25–1	0.25	1	0.5–1	1	1
	VC	0.25–1	0.25	0.5	0.25–1	0.25	0.25	0.25–1	0.25	0.5
Clove	BM	0.25–1	0.25	1	0.25–1	0.25	1	0.125–1	0.25	1
	VC	0.25–1	0.5	0.5	0.25–0.5	0.5	0.5	0.06–1	0.06	0.25
Pine	BM	0.03–0.06	0.06	0.06	0.0075–0.5	0.015	0.25	0.015–0.5	0.03	0.5
	VC	0.5–1	1	1	0.5–1	1	1	0.5–1	1	1
Sage	BM	0.5–1	1	1	0.5–1	1	1	0.5–1	1	1
	VC	0.06	0.06	0.06	0.125–0.25	0.125	0.25	0.06	0.06	0.06
Lemon balm	BM	0.5–1	1	1	0.5–1	0.125	1	0.06–0.5	0.25	0.25
	VC	0.015–0.03	0.015	0.015	0.015–0.06	0.03	0.06	0.0038–0.015	0.015	0.015
Lavender	BM	0.5–1	1	1	0.5–1	0.25	1	0.125–1	0.25	1
	VC	0.0019–0.06	0.06	0.06	0.0075–0.06	0.03	0.06	0.015–0.06	0.03	0.06
Fluconazole	BM	0.5- > 64	0.50	4.00	0.125- > 64	4.00	64.00	0.25- > 64	2.00	16
Voriconazole	BM	0.008–8	0.06	0.12	0.008–8	0.25	4.00	0.032–8	0.12	2.00

Results were obtained from 3 independent experiments performed in duplicate and expressed as modal results

**Table 2:** Minimum inhibitory concentration (MICs) of EOs components (%, v/v) by the broth microdilution (BM) and vapour contact (VC) methods

Components	Methods	*C.albicans* (*n* = 26)			*C.glabrata* (*n* = 10)			*C.tropicalis* (*n* = 10)		
		Range	MIC 50	MIC 90	Range	MIC 50	MIC 90	Range	MIC 50	MIC 90
Carvacrol	BM	0.06–0.5	0.125	0.125	0.06–0.25	0.125	0.25	0.25–0.5	0.25	0.25
	VC	0.0038	0.0038	0.0038	<0.0019	<0.0019	<0.0019	<0.0019–0.0038	<0.0019	<0.0019
Eugenol	BM	0.25	0.25	0.25	0.125–0.25	0.125	0.25	0.25–0.5	0.25	0.25
	VC	0.125–0.5	0.125	0.125	0.06–0.25	0.125	0.125	0.125–0.25	0.125	0.125
Linalool	BM	0.125–1	0.25	0.25	0.125–1	0.5	0.25	0.25–0.5	0.25	0.25
	VC	0.0075	0.0075	0.0075	0.0075–0.03	0.015	0.03	0.0015–0.03	0.03	0.03
Linalyl acetate	BM	1	1	1	1	1	1	1	1	1
	VC	1	1	1	1	1	1	1	1	1
Thymol	BM	0.06–0.125	0.06	0.06	0.06–0.25	0.125	0.125	0.125–0.5	0.125	0.125
	VC	0.0038	0.0038	0.0038	0.0019–0.0038	0.0019	0.0019	<0.0019	<0.0019	<0.0019
α-Pinene	BM	0.06–0.125	0.06	0.06	0.06–0.125	0.06	0.125	0.125–0.5	0.125	0.125
	VC	0.25–0.5	0.25	0.5	0.25–0.5	0.25	0.5	0.25–1	0.5	0.5

Results were obtained from 3 independent experiments performed in duplicate and expressed as modal results

As regard BM, thyme red and pine EOs showed the best activity against all strains tested, though *C.albicans* proved to be more susceptible than *C.glabrata* and *C.tropicalis* to pine oil (MIC_50_ - MIC_90_ = 0.06 %, v/v). On the contrary, fennel, clove, sage and lavender EOs showed the highest MICs (Table 1), whereas lemon balm oil displayed a weak activity against both *C.tropicalis* (MIC_50_ - MIC_90_ = 0.25 %, v/v) and *C. glabrata* (MIC_50_ - MIC_90_ = 0.125–1 %, v/v, respectively).

Interestingly, the MIC values obtained with VC were lower than those in liquid medium for all the EOs tested, except for pine. Specifically, thyme red was the oil with the highest activity (MIC_90_ < 0.0038 %, v/v) against *C.albicans*, followed by lemon balm, lavender, and sage (Table 1).

The different antifungal activity in liquid and vapour phase could be due to the characteristics of EOs such as high hydrophobicity and volatility. In fact, when added to a medium, the EO distributes more or less into the aqueous phase depending on its relative hydrophobicity. In the liquid phase, the activity depends upon the diffusibility and solubility of the EOs in the medium while in the vapour assay it depends upon the volatility [2].

MIC values showed that EOs activity is higher than that obtained with the conventional antifungal drugs tested against *C. glabrata* e *C. tropicalis*, both resistant to fluconazole and voriconazole (fluconazole MIC90 = 64 and 16 μg/mL, respectively and voriconazole MIC90 = 4 and 2 μg/mL, respectively) (Table 1).

The data of the present study can be explained because of chemical composition of EOs, we had already reported in a previous study [8]. These EOs contained the following major components determined by gas chromatography–mass spectrometry: thymol (26.5 %, v/v), ρ-cymene (16.2 %, v/v), limonene (13.2 %, v/v), α-pinene (13.2 %, v/v), carvacrol (7.8 %, v/v) in thyme red; anethole (72.1 %, v/v), fenchone (14.2 %, v/v) in fennel; eugenol (77.5 %, v/v), eugenyl acetate (7.6 %, v/v) in clove; α-pinene (55.7 %, v/v), β-pinene, (10 %, v/v), limonene (9.7 %, v/v) in pine; cis-thujone (29.4 %, v/v), camphor (22.6 %, v/v), 1,8 cineole (7.7 %, v/v) in sage; citronellal (29.4 %, v/v), limonene (22.6 %, v/v), geranial (8.8 %, v/v) in lemon balm; linalool (41.9 %, v/v), linalyl acetate (32.7 %, v/v) in lavender. The EOs tested in this study showed the presence of significant amounts of monoterpenes, mainly represented by thymol, α-pinene, and linalool compounds, in accordance with previous published data even if with different percentile [21] (Table 2). Carvacrol and thymol exerted an interesting anti-Candida in vitro activity both by BM and VC, similar to previous findings [22–24], even against fluconazole-resistant *C. glabrata* and *C. tropicalis* strains. α-pinene showed a better activity by BM than VC; conversely, linalyl acetate showed the lowest activity against all strains tested both by two methods. Generally, MFCs were one or more concentrations higher than MICs (Table 3), suggesting a fungicidal activity of the EOs at low concentrations against yeasts cells, probably due to their related components, such as terpenoids and phenolics known for their broad-spectrum antimicrobial activity [25, 26]. However, the mechanisms behind the antifungal activity of EOs are not fully understood.

**Table 3:** Minimum fungicidal concentration (MFC) of EOs (%, v/v) and their components (%, v/v) in comparison with drugs (μg/mL)

	*C.albicans* (*n* = 26)	*C.glabrata* (*n* = 10)	*C.tropicalis* (*n* = 10)
	MFC	MFC	MFC
Thyme red	0.06-0.25	0.06-0.25	0.06-0.5
Fennel	0.5- > 1	0.25- > 1	1- > 1
Clove	0.25- > 1	0.25- > 1	0.5- > 1
Pine	0.03-0.125	0.03-0.5	0.06-1
Sage	>1	>1	>1
Lemon balm	>1	>1	>1
Lavender	>1	>1	>1
Carvacrol	0.125-1	0.25-1	0.25-1
Eugenol	0.5	0.25	0.5
Linalool	0.5	0.5	1
Linalyl acetate	1- > 1	1- > 1	1- > 1
Thymol	0.125-0.25	0.25	0.25-0.5
α-Pinene	0.5-1	0.5-1	1
Fluconazole	2 - >64	0.5 - >64	0.5- > 64
Voriconazole	0.06-8	0.12-8	0.12-8

Results were obtained from 3 independent experiments performed in duplicate and expressed as modal results

High antifungal activity of examined EOs, also against antibiotic-resistant isolates is according to recent evidence of some authors [20, 27, 28]. However, it is difficult to compare the data with the literature because the antimicrobial activity of EOs and their components are influenced by the several factors including chemical compositions and experimental conditions.

The composition of EOs varies significantly because of plant different species and chemotypes, geographical origin, season and extraction procedure [29]. In this context, Tampieri et al. [30] studied a *T. vulgaris* EO based on carvacrol (41.33 %), p-cymene (17.53 %) and thymol (5.34 %) and observed a fungistatic activity of the EO tested [30]. Conversely, we studied a *T. vulgaris* EO with different composition; in fact, thymol was 26.5 %, p-cymene 16.2 % and carvacrol 7.8 % and we observed a fungicide activity of the EO tested.

Regarding experimental conditions, it is important to underline that EOs antimicrobial activity data depend on the methodology used, the variability of which includes factors such as inoculum size, medium used, and use of sealants, surfactants and solvents such as Tween, dimethylsulphoxide and ethanol. In part, these may explain the differences in results obtained by different research groups [31].

Results obtained in this study highlight the activity of the main compounds, thymol in thyme red oil (26.5 %) and α-pinene in pine oil (55 %). However, some data suggest that components presented in small amounts in EOs, such as carvacrol, also could play an important role in antimicrobial activity due to the possible synergistic action with other components [24, 25, 32, 33].

Thymol, known to be lipophilic, together with carvacrol can enter between the fatty acyl chains making up membrane lipid bilayers, thus altering the fluidity and permeability of cell membranes [34].

Some authors indicate that this action on fungi, particularly on *C. albicans*, affects the regulation and function of important membrane-bound enzymes that catalyze the synthesis of a number of major cell wall polysaccharide components, such as β-glucans, chitin and mannan, thus disturbing cell growth and envelope morphogenesis [26].

Our data on thymol are in agreement with those of Fontenelle et al. [7] who demonstrated its potent antimicrobial activity against *C. albicans* with MIC = 39 μg/mL. On the contrary, our data do not support those of Zore et al. [35], who reported the considerable activity of linalyl acetate against 39/48 yeast isolates with MIC = 0.064 % (v/v), but they are in agreement with D’Auria et al. against *C. albicans* (Table 2) [7, 35, 36].

In accordance with our previous findings against clinical filamentous fungi and since active compounds of EOs are highly volatile, EOs possess high antimicrobial activity in vapour phase [8].

Phenols, such as thymol and carvacrol, are among the most active natural antioxidants and antimicrobials found in EOs [2, 7]. However, due to their poor water solubility and the requirement for high concentrations to reach a therapeutic effect, the efficiency of these compounds in treatment is limited. It is important to emphasize that thymol is a smaller and more volatile molecule than the ether-containing eugenol from clove. According to Suhr et al. [29], thyme was also generally more effective than clove in the volatile experiment (Table 2) [29].

## Conclusions

Data reported in this study show a good activity of EOs, mainly thymus red and pine oils, against *Candida* spp., including fluconazole/voriconazole resistant strains. This activity could be related to main components of EOs, such as carvacrol, thymol, and α-pinene that exerted a significant antifungal action.

Overall, our experimental data give substantial support to previous empirical evidence or literature data on the anticandidal activity of different EOs. These data also encourage adequately controlled and randomized clinical investigations, including different screening methods and action mechanisms studies for future applications: i.e. a volatile screening method should be employed for fumigation or active packaging purposes. The use in vapour phase could have additional advantages without requiring direct contact, resulting in simple and convenient environmental application such as in hospital, and/or in school.

## Abbreviations

BM: Broth microdilution method
EOs: Essential oils
MFC: Minimal fungicidal concentration
MIC: Minimum inhibitory concentration
VC: Vapour contact assay
WT: Wild type
